# Gastrocolic fistula caused by transverse colon cancer: a case report

**DOI:** 10.1186/s40792-023-01590-2

**Published:** 2023-01-23

**Authors:** Tomoyuki Sugi, Masanao Kurata, Tomoaki Furuta, Osamu Ishibashi, Satoshi Inagawa, Hiroyuki Ariga, Junya Kashimura, Hitomi Kawai, Norio Takayashiki, Tatsuya Oda

**Affiliations:** 1 Department of Surgery, Mito Kyodo General Hospital, 3-2-7 Miya-Machi, Ibaraki, 310-0015 Japan; 2 Department of Gastrointestinal and Hepato-Biliary-Pancreatic Surgery, Faculty of Medicine, University of Tsukuba, 2-1-1 Tennodai, Tsukuba, Ibaraki 305-8576 Japan; 3 Department of Internal Medicine, Mito Kyodo General Hospital, 3-2-7 Miya-Machi, Ibaraki, 310-0015 Japan; 4 Department of Diagnostic Pathology, Mito Kyodo General Hospital, 3-2-7 Miya-Machi, Ibaraki, 310-0015 Japan

**Keywords:** Gastrocolic fistula, Colon cancer, En bloc resection

## Abstract

**Background:**

A gastrocolic fistula is an unusual communication between the colon and the stomach. Although colon cancer is the most common malignant cause of gastrocolic fistula in the Western world, the incidence of gastrocolic fistula due to colon cancer is 0.3% in operated cases.

**Case presentation:**

A 68-year-old man presented with anorexia, general malaise, weight loss, and vomiting of fecal matter. Investigations revealed that the patient had a large nonmetastatic splenic flexure tumor that was diagnosed as colon cancer and had invaded the stomach and pancreas. An upper gastrointestinal series confirmed a gastrocolic fistula. Left hemicolectomy, distal gastrectomy, distal pancreatectomy, and splenectomy were performed. Histology revealed transverse colon cancer, which was UICC stage (8th edition) pT4bN1bcM0 pStage IIIC. Adjuvant chemotherapy was not performed. There was no recurrence or metastasis one year after surgery.

We reviewed 17 cases including our case of a gastrocolic fistula caused by colon cancer. Neoadjuvant chemotherapy was not given to any of the patients, and en bloc resections were conducted in all cases. Adjuvant chemotherapy was given to almost all of the patients. There was no recurrence or metastasis.

**Conclusions:**

For gastrocolic fistula caused by advanced colon cancer, secure en bloc surgical resection was the initial treatment in all 17 reported cases including the present case, and adjuvant chemotherapy may contribute to a better prognosis.

## Background

A gastrocolic fistula is a pathologic communication between the colon and stomach. Both benign and malignant etiologies can cause gastrocolic fistulas [1]. The benign causes include gastric ulcers, Crohn’s disease, diverticulitis, cholecystitis, pancreatitis, tuberculosis, and the use of steroids or NSAIDs [2–4]. The reported malignant causes include gastric, colon, and pancreatic tumors, metastasis of lung cancers, and lymphoma [5, 6]. The incidence of gastrocolic fistula that occurs secondary to colon cancer is reported to be ten out of 3200 colon cancer patients who have had surgery [7]. We report a case of a gastrocolic fistula caused by transverse colon cancer and discuss the management through a literature review.

## Case presentation

A 68-year-old man visited our hospital because of anorexia, general malaise, and vomiting of fecal material. Although he had been aware of the symptoms for 3 months, he did not go to the hospital. His weight decreased by 12 kg over a period of 3 months, and his body mass index was 22.0 kg/m^2^ (174.0 cm tall and 66.5 kg weight). The patient had no significant medical history. Laboratory data showed that the patient had severe anemia (hemoglobin: 4.0 g/dl) and malnutrition (albumin: 2.7 g/dl). The tests for tumor markers revealed that the patient’s carbohydrate antigen 19–9 was 8.0 U/ml and his carcinoembryonic antigen was 202.4 ng/ml.

**Fig. 1:**
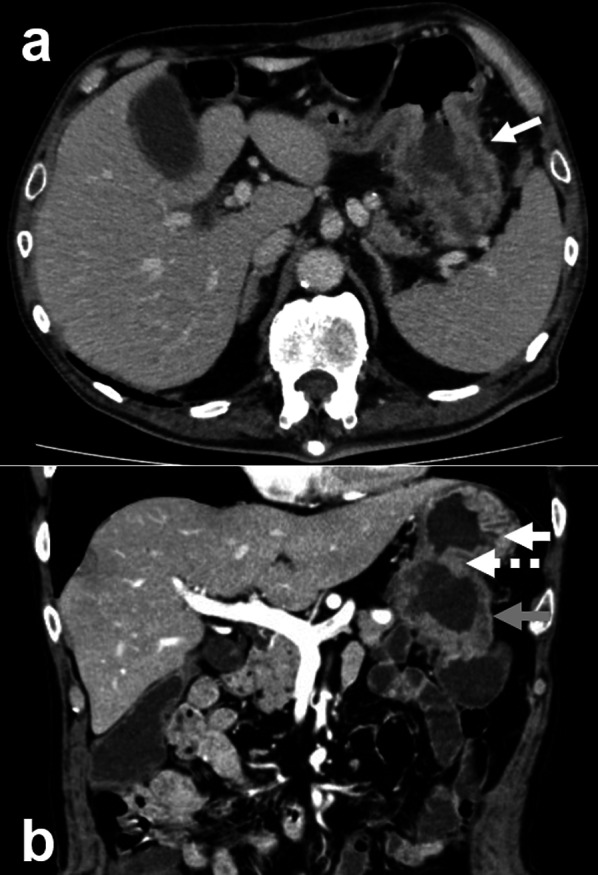
**a** Computed tomography revealed a tumor in the splenic flexure of the colon, and the tumor had invaded the greater curvature of the stomach and the tail of the pancreas. The white arrows indicate gastrocolic fistula. The spleen was enlarged, and there was suspicion of tumor invasion to the splenic vein. **b** The tumor was mainly located in the splenic flexure of the colon, and except for the greater curvature where the tumor invaded, there were no obvious abnormalities in the stomach. The white, white dotted, and grey arrows indicate the stomach, gastrocolic fistula, and splenic flexure of the colon, respectively

**Fig. 2:**
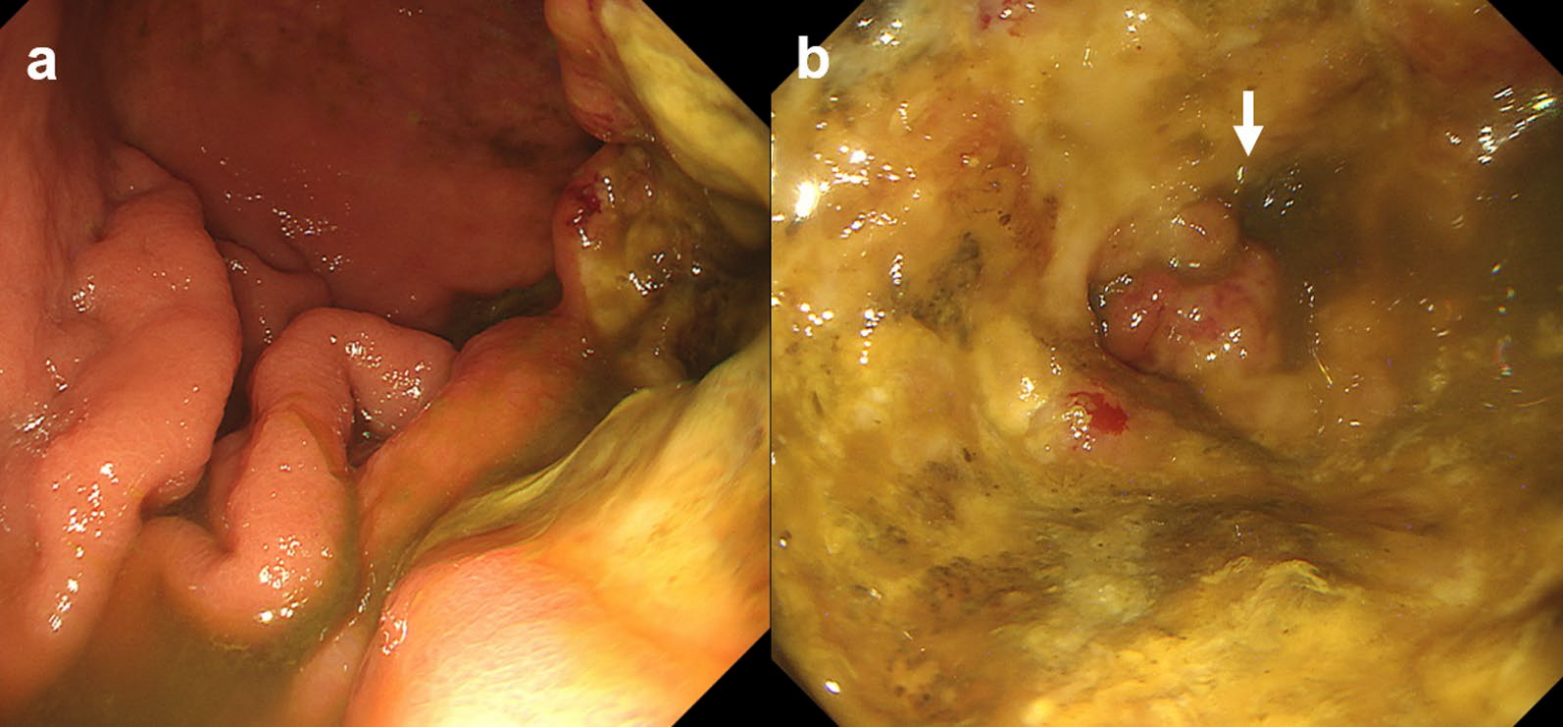
**a** The upper gastrointestinal endoscopy showed a cratered gastric ulcer in the posterior wall of the gastric body. **b** There was fluid with fecal material in the stomach. The white arrow indicates the hole in the gastric body, which raised suspicion for a possible connection to the colon

**Fig. 3:**
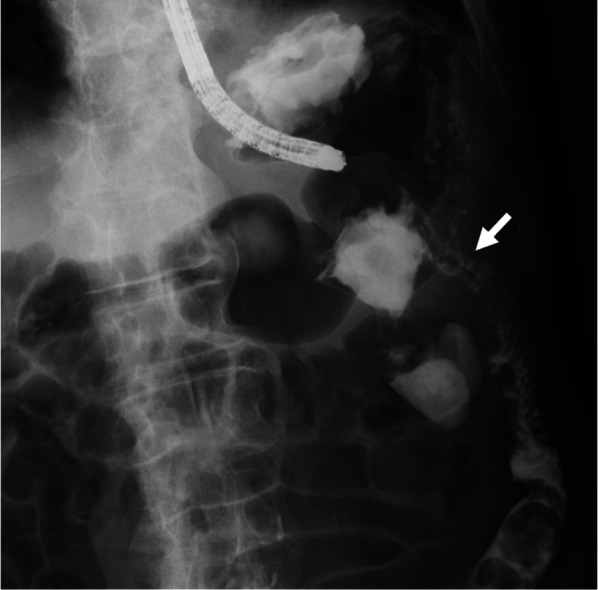
Upper gastrointestinal series confirmed a fistulous path to the colon from the stomach. The white arrow indicates gastrocolic fistula

A computed tomography (CT) scan of the abdomen with contrast revealed a tumor in the splenic flexure of the colon, and the tumor had invaded the greater curvature of the stomach and the tail of the pancreas (Fig. 1a). The tumor was mainly located in the splenic flexure colon, and except for the greater curvature where the tumor invaded, there were no obvious abnormalities in the stomach (Fig. 1b). The spleen was enlarged and there was suspicion of tumor invasion to the splenic vein (Fig. 1a). There was no distant metastasis, including peritoneal dissemination. Upper gastrointestinal endoscopy showed a large cratered gastric ulcer in the posterior wall of the gastric body (Fig. 2a). There was fluid in the patient’s stomach that contained fecal material, as well as a hole in the gastric body, which raised suspicion for possible connection to the colon (Fig. 2b). A biopsy of the hole revealed a moderately differentiated adenocarcinoma. An upper gastrointestinal series, performed with iodinated contrast, confirmed that there was a fistulous path to the transverse colon from the stomach (Fig. 3). We diagnosed a gastrocolic fistula due to clinical T4bN1bM0 Stage IIIC transverse colon cancer according to the 8th edition UICC guidelines [8].

**Fig. 4:**
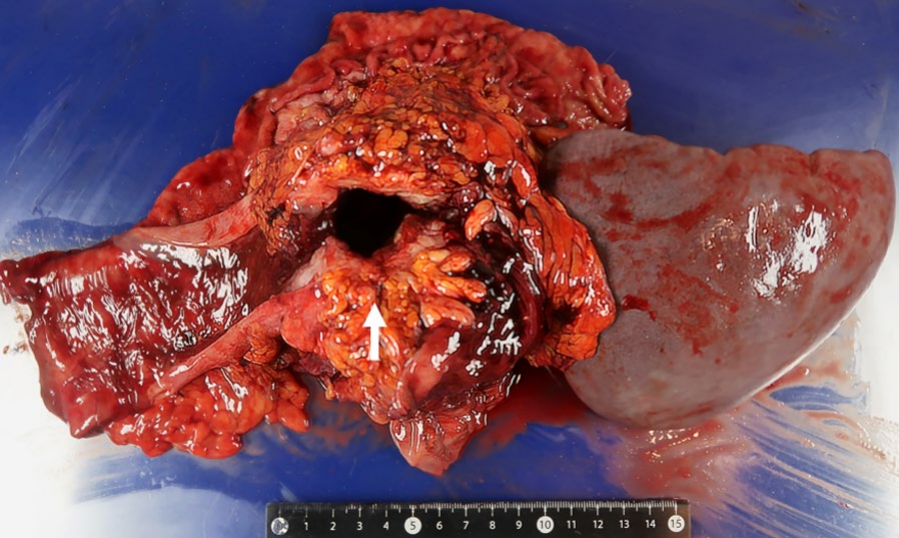
A radical en bloc resection involving a left hemicolectomy, distal gastrectomy, distal pancreatectomy, and splenectomy was conducted. The white arrow indicates the gastrocolic fistula that was created by the transverse colon tumor

**Fig. 5:**
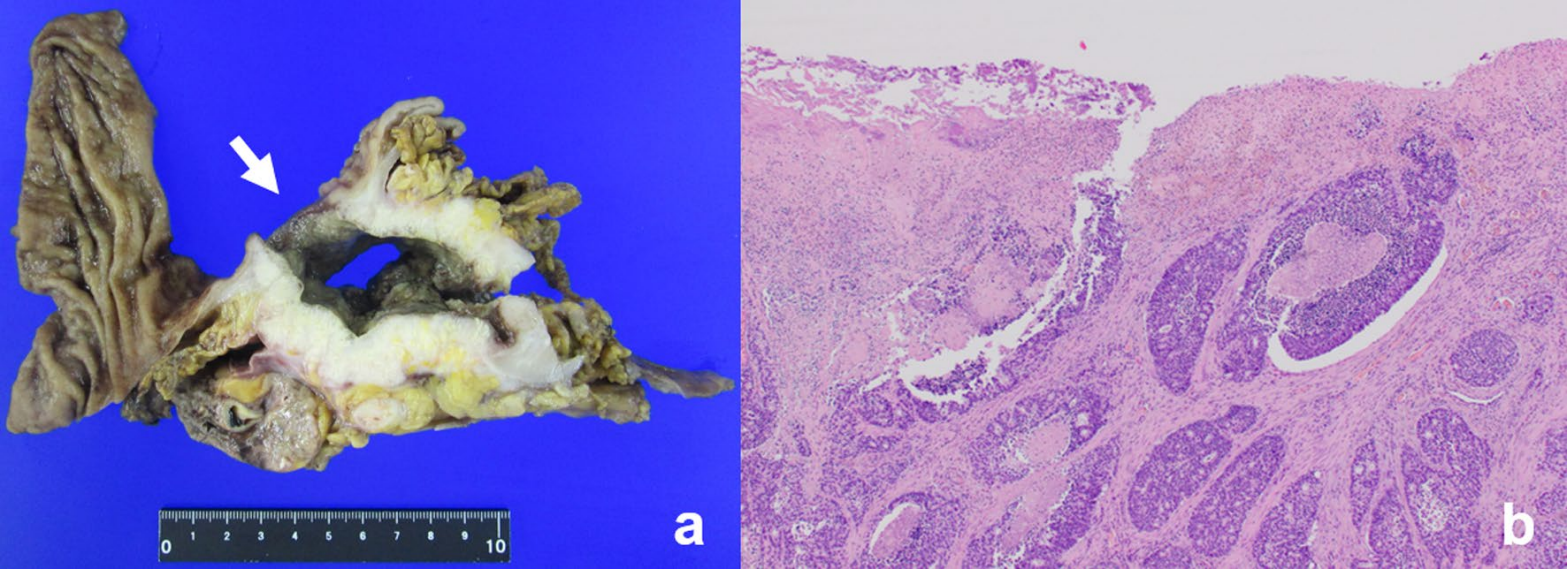
**a** The white arrow indicates the gastrocolic fistula that surrounded the tumor. **b** The surface of the fistula was covered with tumor cells

We considered that the tumor could be completely removed if the invaded or adjacent organs were also resected. A radical en bloc resection, involving a left hemicolectomy, distal gastrectomy, distal pancreatectomy, and splenectomy was conducted. We could not avoid resection of the pancreas because the tumor strongly adhered to it, and there was a suspicion of tumor invasion. The spleen was also excised because the splenic vein was adhered to the tumor, and there was a suspicion of tumor invasion. The specimen showed that the tumor of the transverse colon had invaded the stomach and created a gastrocolic fistula (Fig. 4). Histology revealed that the tumor originated from the colon and had invaded the stomach. A gastrocolic fistula surrounded the tumor, and the surface of the fistula was covered with tumor cells (Fig. 5). The pancreas and the spleen were histologically free of the tumor. The histology also revealed the presence of lymph node metastasis in three out of the 55 retrieved lymph nodes. All metastatic lymph nodes were paracolic. The surgical margins were free of tumor cells. He was diagnosed with pathological T4bN1bM0 stage IIIC transverse colon cancer according to the 8th edition UICC guidelines [8].

Postoperatively, the patient developed delayed gastric emptying. For this complication, conservative management required the patient to eat nothing, and a drip intravenous injection was performed: the patient eventually recovered with a good appetite. The patient recovered his nutritional condition (albumin: 3.6 g/dl) and was discharged on postoperative day 66. Although we recommended adjuvant chemotherapy, the patient refused to receive this treatment. One year after surgery, there was no recurrence or metastasis.

## Discussion

The first case of gastrocolic fistula was reported by Haller in 1775 [7]. Colon cancers are the most common malignant cause of gastrocolic fistula in Western countries, while gastric cancers are the most common malignant cause in Japan [9]. The average age of patients with gastrocolic fistula caused by cancer was reported to be 64.2 years [7]. No unique sex distribution has been noted in patients with gastrocolic fistulas secondary to cancer [5]. Gastrocolic fistulas most commonly occur between the gastric greater curvature and the distal transverse colon because of the proximity of these two structures [7, 10].

Two theories have been proposed for the development of gastrocolic fistulas secondary to colon cancer: the first theory is contiguous growth of the tumor, and the second is chronic ulceration of the primary tumor with the development of peritoneal reaction or organization of exudate, leading to the adherence of adjacent structures and eventual perforation into the lumens of both structures [5]. In our case, the gastrocolic fistula surrounded the tumor and the surface of the fistula was covered with tumor cells. It was possible that the tumor had directly invaded the stomach and that the tumor invasion eventually created the fistula (Fig. 5).

The main clinical findings in patients with gastrocolic fistulas are weight loss, pain, vomiting of fecal material and diarrhea, and the frequencies have been reported to be 91%, 64%, 45% and 36%, respectively [7]. Laboratory tests often reveal malnutrition, anemia, and acute or chronic electrolyte loss [5, 7]. Referring to our case, the patient complained of weight loss and fecal vomiting, and his laboratory tests were consistent with severe anemia and decreased serum albumin levels.

In the past, when CT scans were not widely used, the most reliable modality for the diagnosis of gastrocolic fistula was barium enema, which has a specificity of 90–100% [11]. Now that CT scans have become common and the technology has improved, gastrocolic fistula is often discovered by CT scans. CT scans are also of great value for assessing metastasis and the local invasion of the primary tumor [11]. Upper gastrointestinal imaging, such as barium enema and iodinated contrast, can directly visualize gastrocolic fistula. Iodinated contrast with an upper gastrointestinal series instead of a barium enema was performed in our case, and the results were sufficient to make a diagnosis. Upper gastrointestinal endoscopy allows direct visualization of the hole in the gastric body and can be used to determine if a biopsy is necessary to establish a pathologic diagnosis [3].

If there is no metastasis, surgery is the basic treatment for colon cancer accompanied by a gastrocolic fistula. The American Society of Colon and Rectal Surgeons clinical practice guidelines strongly recommend that colon cancer with adherent or grossly involved adjacent organs should be treated with an en bloc resection, and not a separated resection [12]. The guidelines state that it is impossible to intraoperatively distinguish between malignant and inflammatory adhesions during surgery [12]. Referring to our case, distal pancreatectomy and splenectomy were conducted because the tail of the pancreas and the splenic vein were strongly adhered to the tumor although there was no histological invasion to the pancreas or the spleen.

We searched for the keywords “gastrocolic fistula” and “colon cancer” in PubMed, and we found 16 surgical resection cases written in English that were included in our evaluation here, in addition to our case (Table 1) [1–3, 6, 9, 10, 13–21]. The ratio of men to women was 9:7. The mean age of the patients was 56.1 years. The tumor location was in the transverse colon or splenic flexure in 16 out of the 17 cases. Weight loss, vomiting of the fecal material, and diarrhea were seen in 15 out of 17 patients (88.2%), 8 out of 17 patients (47.1%), and 10 out of 17 patients (58.8%), respectively. The adjacent organs that were involved with the combined resections were the pancreas in 7 out of 17 cases, the spleen in 8 out of 17 cases, the small intestine in 6 out of 17 cases, and other organs (left diaphragm and left adrenal) in 2 out of 17 cases.

**Table 1:** Cases of surgical resection of gastrocolic fistulas secondary to colon cancer

Author	Age/sex	Tumor location	Chief complaints			Resection	Combined resection organs				pN	cM	Neoadjuvant chemotherapy	Adjuvant chemotherapy	Prognosis
			Weight loss	Fecal vomiting	Diarrhea		Pancreas	Spleen	Small intestine	The others					
Our case	68/M	Splenic flexure	●	●		En bloc	●	●			1b	0	No	No	No recurrence
Ammori [6]	54/M	Splenic flexure	●	●		En bloc		●			1a	0	No	FOLFOX	N/A
Chime. [1]	85/F	Transverse colon	●	●		En bloc					0	0	No	Yes	N/A
Bacalbasa [13]	61	Transverse colon	●			En bloc	●		●		1a	0	No	5-FU	No recurrence
Orosey [10]	65/M	Transverse colon	●	●	●	En bloc	●	●	●		N/A	0	No	Yes	N/A
Fernández [2]	48/M	Splenic flexure	●		●	En bloc			●	Left diaphragm	0	0	No	CAPOX	No recurrence
Huttenhuis [3]	47/F	Transverse colon	●		●	En bloc	●	●			2b	0	No	CAPOX	No recurrence
Harkin [14]	51/F	Splenic flexure	●	●		En bloc		●			N/A	0	No	5-FU	N/A
Wang [15]	54/M	Transverse colon	●		●	En bloc			●		N/A	0	No	UNK	N/A
Tejedor [16]	49/M	Splenic flexure	●	●	●	En bloc		●	●		0	0	No	Yes	No recurrence
Imai [17]	60/F	Splenic flexure	●		●	En bloc	●	●			1	0	No	UFT/UZEL	No recurrence
Matar [18]	52/M	Splenic flexure	●		●	En bloc					0	0	No	Capecitabine	N/A
Forshaw [19]	24/F	Transverse colon	●	●	●	En bloc			●		0	0	No	5-FU	No recurrence
Lee [20]	41/M	Splenic flexure			●	En bloc					N/A	0	No	UNK	N/A
Lee [20]	73/F	Splenic flexure				En bloc	●	●		Left adrenal	N/A	0	No	UNK	N/A
Singh [21]	49/M	Descending colon	●	●	●	En bloc					1a	0	No	UNK	N/A
Matsuo [9]	72/F	Transverse colon	●			En bloc					0	0	No	UNK	No recurrence

pT and pN are according to the UICC 8th edition

*N/A* not available, *CAPOX* capecitabine and oxaliplatin, *FOLFOX* folinic acid, 5-fluorouracil, and oxaliplatin, *5-FU* 5-fluorouracil and folinic acid, *UFT/UZEL* uracil/ftorafur plus leucovorin

An en bloc resection including adjacent organs if necessary was achieved in all cases and separation surgery was not conducted in any of the patients. These factors may have contributed to the reason why there was no recurrence or death in the patients. Hunter et al. [22] reported a significantly higher 5-year survival (61% vs. 23%, *P* = 0.03) after en bloc resection of colon cancer compared with colectomy with separation of adherent organs; the latter approach was associated with an unacceptably high local recurrence rate (69% vs. 18%, *P* = 0.002). For colon cancer accompanying gastrocolic fistula, en bloc resection of the colon and stomach is especially desirable because the lumen of the fistula may be covered with tumor cells [5]. In addition, surgeons should not hesitate to resect adjacent organs in addition to the colon and stomach if necessary in patients with colon cancer accompanying gastrocolic fistula.

Neoadjuvant chemotherapy is sometimes given for locally advanced colon cancer. Dehal et al. [23] reported that neoadjuvant chemotherapy improved survival in patients with clinical T4b colon cancer. Our study reveals that there were no cases for which neoadjuvant chemotherapy was used to treat colon cancer with a gastrocolic fistula. There is a possibility that the general condition of patients with gastrocolic fistula secondary to colon cancer may be too severe, for instance, they may be too malnourished, to receive neoadjuvant chemotherapy, as in our case.

Information about adjuvant chemotherapy was obtained in 12 out of 17 patients. Adjuvant chemotherapy was performed in almost all of the patients (11 out of 12). Five out of 11 patients were treated with adjuvant chemotherapy, even if the patient had no lymph node metastases. The European Society for Medical Oncology (ESMO) consensus guidelines define patients with T4 colon cancer as a high-risk group and recommend adjuvant chemotherapy [24]. Adjuvant chemotherapy may be considered the preferred treatment for colon cancer involving a gastrocolic fistula. The regimen is mainly composed of fluoropyrimidine and platinum-containing drugs.

## Conclusions

Secure en bloc surgical resection of both the stomach and colon is mandatory for gastrocolic fistula caused by advanced colon cancer, and adjuvant chemotherapy may be advisable if feasible for prolongation of the prognosis.

## Data Availability

No additional data.

## Abbreviations

CT: Computed tomography
ESMO: European Society for Medical Oncology
